# High Incidence of Multiple-Drug-Resistant Pheromone-Responsive Plasmids and Transmissions of VanA-Type Vancomycin-Resistant *Enterococcus faecalis* between Livestock and Humans in Taiwan

**DOI:** 10.3390/antibiotics12121668

**Published:** 2023-11-27

**Authors:** Haruyoshi Tomita, Jang-Jih Lu, Yasuyoshi Ike

**Affiliations:** 1 Department of Bacteriology, Gunma University Graduate School of Medicine, Maebashi 371-8511, Gunma, Japan; 2 Laboratory of Bacterial Drug Resistance, Gunma University Graduate School of Medicine, Maebashi 371-8511, Gunma, Japan; 3 Department of Laboratory Medicine, Linkou Chang Gung Memorial Hospital, Taoyuan 333, Taiwan; janglu45@gmail.com; 4 Department of Medicine, College of Medicine, Chang Gung University, Taoyuan 333, Taiwan

**Keywords:** VRE, VanA, *E. faecalis*, pheromone-responsive plasmid, Taiwan, livestock

## Abstract

A total of seventy VanA-type vancomycin-resistant enterococci (VRE) isolates obtained in Taiwan in the early 2000s were retrospectively characterized. Forty isolates were obtained from human patients and thirty from livestock. Of these VRE isolates, twenty-three (57.5%) of the human VRE and thirty (100%) of the livestock VRE were *Enterococcus faecalis*, and the remaining seventeen (42.5%) of the human VRE were *E. faecium*. Of the 53 *E. faecalis* isolates, twenty-two (96%) of the human VRE and thirty (100%) of the livestock VRE exhibited a high level of resistance to vancomycin and sensitivity to teicoplanin. They also had three amino acid substitutions in the N-terminal region of the deduced VanS sequence. The vancomycin resistance of all of the 22 human isolates, and 20 of the 30 livestock isolates, transferred to *E. faecalis* FA2-2 at a frequency of 10^−5^ to 10^−3^ per donor cell in broth. Each of the transconjugants responded to *E. faecalis* pheromone (i.e., *E. faecalis* FA2-2 culture filtrate), indicating that the conjugative plasmids were pheromone-responsive plasmids. Three of the conjugative plasmids originated from human isolates, and five plasmids from livestock isolates were corresponded and classified as type A plasmid. Two plasmids originated from human isolates and six plasmids from livestock isolates were corresponded and classified as type B plasmid. *E. faecalis* FA2-2 containing either the type A or type B plasmid responded to the synthetic pheromone cAD1. The type A and type B plasmids transferred between *E. faecalis* FA2-2 and JH2SS at a frequency of about 10^−2^ per donor cell and conferred vancomycin, bacitracin, and erythromycin resistances. The complete DNA sequence of the representative type A plasmid pTW9 (85,068 bp) showed that the plasmid carried a Tn*1546*-like element encoding *vanA*-type resistance, erythromycin resistance (*ermB*), and bacitracin resistance (*bcrABDR*). The plasmid contained the regulatory region found in the pheromone-responsive plasmid and encoded the genes *traA*, *traD* and *iad1*, which are the key negative regulatory elements, and *traE1*, a key positive regulator of plasmid pAD1, indicating that plasmid pTW9 was pAD1-type pheromone-responsive plasmid. PFGE analysis of SmaI-digested chromosomal DNAs showed that several *E. faecalis* strains harboring an identical type A pheromone-responsive plasmid were indistinguishable, and that these were identified both in human and livestock isolates, indicating the transmissions of the VRE strains between livestock and humans. These data showed that the multiple-drug-resistant pheromone-responsive conjugative plasmids have been widely spread in both human and livestock VRE, and there was high potential for transfers of VRE from food animals to humans in Taiwan in the early 2000s.

## 1. Introduction

VanA-type vancomycin-resistant enterococci (VRE) strains are the most common VRE strains to be acquired in the world [1,2]. VRE have been predominantly identified in *Enterococcus faecium* all over the world, although they have been frequently found in *E. faecalis* in the East Asian and Oceanic countries [1,3,4]. The typical VanA-type strain is defined as having a high-level resistance to vancomycin and teicoplanin [5,6]. These VanA determinants are composed of operon gene clusters and consist of *vanRS* and *vanHAXYZ,* which are the regulatory genes and the structural genes, respectively [5]. The VanA determinants are encoded on transposon Tn*1546* or a Tn*1546*-like element [7,8,9].

Vancomycin resistance can be disseminated both by the clonal spread of resistant enterococci and by the horizontal transmission of the resistance genes. Horizontal transmission of vancomycin resistance can be readily observed as a result of plasmid transfer of a conjugative plasmid carrying Tn*1546* when VanA-type *E. faecium* mates on solid surfaces [6,8]. Three types of highly efficient conjugative plasmids that transfer highly efficiently during broth mating have been identified in *E. faecium* and *E. faecalis* [10,11,12]. The pheromone-independent pMG1-like conjugative plasmids and pELF1-like conjugative linear plasmids, which transfer highly efficiently between enterococcal strains during broth mating [13,14,15], are commonly found in *E. faecium* [16,17,18,19]. Tn*1546* is also present both on the pMG1-like conjugative *E. faecium* plasmid and on the pELF1-like conjugative linear enterococci plasmids [12,15,19,20,21,22].

Most pheromone-responsive plasmids are found in *E. faecalis* [10,23,24]. These plasmids exhibit a narrow host range and transfer between *E. faecalis* strains at a high frequency (10^−1^ to 10^−3^ per donor cell) within a few hours of the onset of broth mating. The plasmids confer a mating response to a small peptide (i.e., a sex pheromone) secreted by potential recipient cells. This mating signal induces the synthesis of a surface aggregation substance that facilitates the formation of mating aggregates. Plasmid-free recipients secrete multiple sex pheromones, each specific for a donor harboring a related pheromone-responsive plasmid. Determinants encoded on pheromone-responsive plasmids include those for hemolysin, bacteriocin, and resistance to UV light and antibiotics, including VanA-type and VanB-type vancomycin resistances [25,26,27,28,29,30,31].

Besides the efficient genetic exchange systems of enterococci, the direct selective pressure of glycopeptides is the largest contributing factor in the selective increase of VRE in different habitats such as healthcare environments, and healthy humans and animals (livestock and animal companions). It is strongly suggested that the use of avoparcin as a growth promoters in livestock feed has resulted in the selective increase of VRE in the human community [32,33]. In Europe, VanA-type VRE strains are widespread among food animals and foods of animal origin. The food chain has been implicated as a possible route for the transmission of VanA-type VRE to humans. Several reports have provided the possibilities for genetic exchange or horizontal transmission between human and animal VRE reservoirs [9,29,34,35], and indistinguishable VRE and *vanA*-containing elements have been found in a turkey sample and a turkey farmer [36]. Previously, we showed that nontypical VanA-type *E. faecalis* strains were frequently isolated from chicken imported to Japan from Thailand [32,37,38]. The VanA-type strains showed high-level resistance to vancomycin and sensitivity (or low-level resistance) to teicoplanin (VanB-like phenotype), and had three amino acid substitutions in the *vanS* gene of the VanA determinants [38]. L50 is converted to V, E54 is converted to Q, and Q69 is converted to H. This type of VanA determinants was also identified in VanA-type VRE from human isolates in Japan and was found in poultry in Europe [37,39]. Surveys of chickens carried out in 1999 and 2000, and a survey of human clinical isolates carried out in 1998, showed high-level resistance to vancomycin and sensitivity to teicoplanin and the presence of the three amino acid substitutions in the *vanS* gene of the VanA determinants [37]. We have also reported that very similar if not identical pheromone-responsive plasmids or identical *vanA* determinants have been found in human and livestock VRE isolates in Korea and showed evidence that the pheromone-responsive plasmid would have transferred to the human-adapted *E. faecalis* strain from animal VRE temporarily colonizing the human intestine [29].

Taiwan, like many European countries, has used avoparcin for 23 years, from 1977 to 2000 as a food additive for growth promotion in food animals [40]. The first human VRE was reported in 1996 in Taiwan. In the early 2000s, the rate of vancomycin resistance among human enterococci was estimated at less than 1%. After the 2000s, VanA-type *E. faecalis* VRE strains rapidly increased and were frequently isolated from chicken and human clinical isolates [41]. In this retrospective study, we characterized the VanA-type *E. faecalis* VRE strains isolated in Taiwan in the early 2000s to clarify the relationship between livestock VRE and human VRE.

## 2. Results and Discussion

### 2.1. VRE Isolates and the Drug Resistances

Forty VRE isolates have been obtained from human clinical isolates, and thirty isolates have been obtained from livestock in Taiwan in the early 2000s. PCR analysis showed that all of the strains encoded the *vanA* gene. Of the forty human clinical isolates, twenty-three (57.5%) were *E. faecalis* and seventeen (42.5%) were *E. faecium*. Twenty-two of the twenty-three *E. faecalis* (excluding TVH240 (Hospital B)) showed high-level resistance to vancomycin and low-level resistance or sensitivity to teicoplanin (Supplementary Table S1). These twenty-two VanB-like phenotype *E. faecalis* strains were used for further analysis to examine the relationship between human VRE isolates and animal VRE isolates since this type of resistance was frequently correlated with the VRE isolates from livestock [32,37,38,39,41].

All of the thirty VRE isolates obtained from livestock were *E. faecalis* strains and showed high-level resistance to vancomycin and sensitivity to teicoplanin (Supplementary Table S1). All thirty isolates were used in further analysis. The VRE isolates were examined for drug resistance. All of the isolates were resistant to more than four drugs investigated in this study, as shown in Supplementary Table S1. The numbers and percentages of VRE isolates resistant to each of the drugs are shown in Table 1. Resistances to ERY, KAN, STR, CHL, TET, and BAC were frequently isolated in both humans and livestock [42,43].

In Taiwan, a variety of antibiotic agents including avoparcin (glycopeptide), macrolides, and bacitracin have been used for food animals (including poultry and pig) as growth promoters more than 20 years, before when most of them have been banned in 2001 [41]. The high incidence of the multiple-drug-resistant VRE isolates from livestock correlated with the usage of the antibiotics was reported [40].

### 2.2. VanS Gene Polymorphism

The nucleotide sequence and deduced amino acid residues for the three amino acid substitutions in the N-terminal region of the deduced VanS sequence were identified in all of the 52 strains (22 human VRE and 30 livestock VRE). L50 had been converted to V, E54 had been converted to Q, and Q69 had been converted to H when compared to the *vanS* gene sequence of Tn*1546*. The substitutions were identical to those found in VanS of VanA-genotype VanB-phenotype VRE isolated from chicken imported to Japan from Thailand [37,38].

### 2.3. PFGE and MLST Analyses of the VRE Isolates

**Figure 1:**
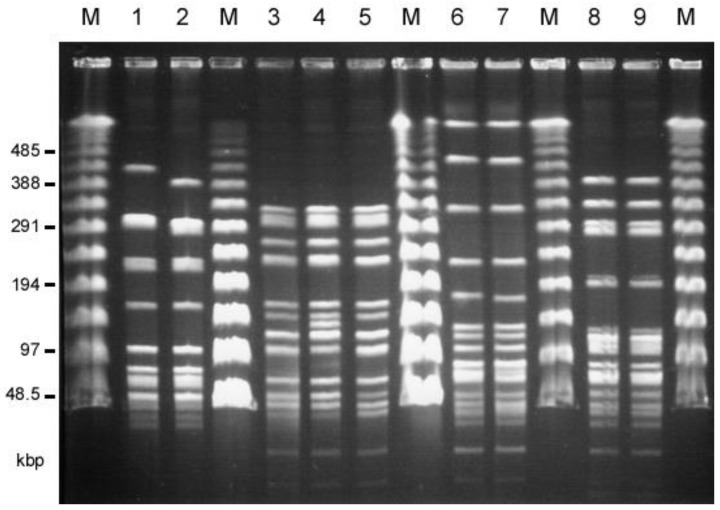
Pulsed-field gel electrophoresis (PFGE) of SmaI-digested total DNAs of human (patients) and livestock VRE isolates that showed almost identical patterns. M, Lambda ladder PFGE marker (New England Biolabs, Beverly, MA, USA); 1, TVH209 (human, hospital G); 2, TVA126 (chicken feather,); 3, TVH208 (human, hospital G); 4, TVH217 (human, hospital D); 5, TVA122 (chicken feather), 6, TVH222 (human, hospital D); 7, TVA117 (pig waste); 8, TVA113 (chicken meat), 9, TVA118 (pig waste).

The twenty-two *E. faecalis* human isolates and thirty *E. faecalis* livestock isolates, which showed the VanB-like phenotypes, were analyzed by PFGE (Supplementary Figure S1 and Figure S2, respectively). The SmaI-digested patterns of the TVH208 and TVH217 strains isolated from among the human isolates, and the TVA118 and TVA124 strains isolated from among the livestock isolates, were almost identical, respectively (Figure 1, Table 2). A comparison of the SmaI-digested patterns of TVH209 and TVA126 from the human and livestock isolates showed that they were almost identical. In addition, TVH208 and TVH217 from the human isolates and TVA122 from livestock isolates were almost identical, as was the pairing of TVH222 and TVA117 from human and livestock isolates, respectively. TVA113 from chicken meat and TVA118 from pig waste also showed very similar patterns (Figure 1, Table 2). Other strains showed different patterns. MLST analysis was performed. TVH209 and TVA126 belonged to ST263 (each of the allele numbers for *gdh*, *gyd*, *pstS*, *gki*, *aroE*, *ept*, and *yqiL* were 41, 2, 18, 10, 16, 2, and 12, respectively), TVH208, 217, and TVA122 belonged to ST264 (each of the allele numbers for *gdh*, *gyd*, *pstS*, *gki*, *aroE*, *ept*, and *yqiL* were 41, 2, 11, 17, 4, 2, and 1, respectively), TVH222 and TVA117 belonged to ST265 (each of the allele numbers for *gdh*, *gyd*, *pstS*, *gki*, *aroE*, *ept*, and *yqiL* were 5, 1, 1, 3, 9, 7, and 6, respectively), and TVA113 and TVA118 belonged to ST266 (each of the allele numbers for *gdh*, *gyd*, *pstS*, *gki*, *aroE*, *ept*, and *yqiL* were 25, 2, 35, 9, 23, 18, and 26, respectively). These results indicated that many of the VRE isolates from humans and livestock, respectively, were different strains. Some groups of identical strains (i.e., TVH209/TVA126, TVH208/TVH217/TVA122, and TVH222/TVA117, respectively) were identified from both the human and livestock environment.

The closely related ST types with the new ST were found in the MLST database (Table 2). ST263 was a single-locus variant of ST33 or ST59, which are originally found in humans and poultry in Europe, respectively [39,44,45]. ST264 is a double-locus variant of ST86, which has an unknown origin. ST265 is a single-locus variant of ST16, grouped into CC16 and found in humans, swine, and poultry [44,46,47]. ST266 is a single-locus variant of ST93, which was originally isolated from healthy human feces in the Solomon Islands [44,47]. The closely related ST types except for the clonal ST16 are not frequently isolated and have no founder [44,46,48]. It seemed that new ST-type isolates (i.e., ST263, ST265, and ST266) would not relate to the previously reported *E. faecalis* strains.

These results indicated the evidence of the transmissions of VanA-type VRE strains between livestock (food animals) and humans in Taiwan.

### 2.4. Conjugative Transfer of Vancomycin Resistance and Analysis of the Conjugative Plasmids

**Figure 2:**
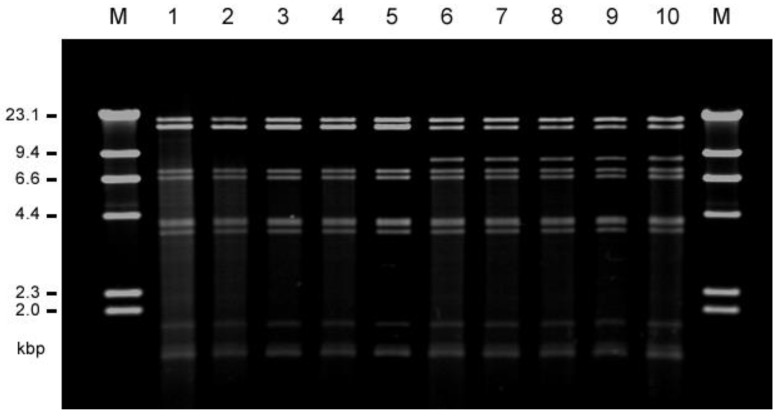
Agarose gel electrophoresis of the vancomycin-resistant pheromone-responsive highly conjugative plasmid DNAs isolated from human (patients) and livestock VRE strains. The restriction endonuclease (EcoRI)-digested plasmid DNAs isolated from each representative transconjugant of FA2-2 were classified into two types, type A (pTW9-like, lines 1 to 5), and type B (pTW24-like, lines 6 to 10). M, HindIII-digested lambda DNA; 1, TVH209 (human, hospital G (pTW9)); 2, TVH216 (human, hospital D); 3, TVA102 (chicken meat); 4, TVA121 (chicken feather); 5, TVA127 (chicken feather), 6, TVH224 (human, hospital D (pTW24, 77,792 bp)); 7, TVH227 (human, hospital C); 8, TVA113 (chicken meat); 9, TVA119 (pig waste); 10, TVA122 (chicken feather).

The conjugative transfer of each of the vancomycin resistance determinants from each of *E. faecalis* VRE isolates to the recipient strain *E. faecalis* FA2-2 or *E. faecium* BM4105RF and BM4105SS was examined. The vancomycin resistance of all of the 22 human isolates and 20 of the 30 livestock isolates transferred to *E. faecalis* FA2-2 at a frequency of 10^−5^ to 10^−3^ per donor cell, but none of them transferred to *E. faecium* strains. Plasmid DNA was prepared from each of the transconjugants, and the EcoRI-digested plasmid DNAs were analyzed by agarose gel electrophoresis (Supplementary Table S1). The plasmid DNAs isolated from each of three transconjugants derived from *E. faecalis* strains TVH209 (hospital G), TVH216 (D), and TVH225 (D) from the human isolates were identical with respect to their EcoRI restriction profiles and classified into type A plasmid. The plasmid DNAs isolated from each of two transconjugants derived from *E. faecalis* strains TVH224 (D) and TVH227 (C) from the human isolates were also identical but different from type A and were classified into type B plasmid. The plasmid DNAs isolated from each of the five transconjugants derived from strains *E. faecalis* TVA102 (chicken meat; CM), 121 (chicken feather; CF), 124 (CF), 126 (CF), and 127 (CF) from livestock isolates corresponded to type A plasmid. The plasmid DNAs isolated from each of the six transconjugants derived from *E. faecalis* TVA104 (CM), 113 (CM), 118 (pig waste; PW), 119 (PW), 122 (CF), and 129 (CF) from the livestock isolates corresponded to type B plasmid, with respect to their EcoRI restriction profiles. Figure 2 shows the representative plasmid DNAs derived from the transconjugants (five type A plasmids and five type B plasmids). Agarose gel electrophoresis analysis showed that the molecular size of type A plasmid was about 7.3 kbp longer than that of type B plasmid (the third EcoRI fragments in Figure 2). The vancomycin resistances of type A and type B plasmid transferred between *E. faecalis* FA2-2 and *E. faecalis* JH2SS at a frequency of about 10^−3^ per donor cell during 4 h of broth mating. Each of the remaining vancomycin-resistant transconjugants from other VRE isolates showed the different plasmid profiles by the agarose gel electrophoresis analysis; therefore, we focused on the widely spread type A and type B plasmids and particularly analyzed them in detail in the present study.

### 2.5. Pheromone Response of the Conjugative Plasmids

The *E. faecalis* FA2-2 transconjugants were examined for pheromone response to induce the mating aggregation (Supplementary Table S1). Of the 22 transconjugants from the 22 human isolates, 11 transconjugants responded to cAD1, 5 transconjugants responded to cOB1, and 6 transconjugants responded to the *E. faecalis* FA2-2 culture filtrate, but they did not respond to the synthetic pheromones. Of the 20 transconjugants from the 30 livestock isolates, 13 transconjugants responded to cAD1, 3 transconjugants responded to cOB1, 4 transconjugants responded to *E. faecalis* FA2-2 culture filtrate, but they did not respond to any synthetic pheromones. *E. faecalis* FA2-2 transconjugants harboring type A or type B plasmid responded to the synthetic pheromone cAD1.

As shown in Figure 1 and Figure 2 and Table 2, TVH209 and the related TVA126 strains both harbored an identical type A plasmid, and TVA113 and the related TVA118 harbored an identical type B plasmid, respectively. Strains TVH208, TVH217, and TVA122, which had a similar PFGE pattern, harbored different plasmids, and TVH208 harbored a pheromone-responsive *vanA* plasmid which responded to a pheromone that differed from the reported pheromones [23,49], TVH217 harbored a non-type A and type B pheromone-responsive *vanA* plasmid which responded to cAD1 pheromone. TVA122 harbored a type B plasmid. TVH222 and TVA117 had a similar PFGE pattern and harbored a pheromone-responsive conjugative *vanA* plasmid that responded to pheromone that differed from reported pheromones, and a non-conjugative *vanA* plasmid, respectively.

### 2.6. Conjugative Transfer of Drug Resistances along with Conjugative Transfer of Vancomycin

The vancomycin-resistant isolates showed multiple-drug resistance. Conjugative transfers of drug resistances along with the transfer of vancomycin were examined in the nine strains selected as representative strains, and the results are shown in Table 2. The transconjugants were grown on selective agar plates containing vancomycin, rifampin, and fusidic acid after broth mating experiments between each of the vancomycin-resistant strains and the recipient strain *E. faecalis* FA2-2 and were examined for their drug resistances. FA2-2 transconjugants harboring the type A plasmid pTW9 or type B plasmid pTW24 exhibited resistance to vancomycin, erythromycin, and bacitracin, and low-level resistance to teicoplanin. The drug resistance levels were (MIC, mg L^−1^) 512, >1024, >256, and 8, respectively. FA2-2 transconjugants of the donor strain TVH208 exhibited resistances to VAN and CHL. The transconjugant of TVH217 exhibited resistances to VAN, ERY, and BAC. The transconjugant of TVH222 exhibited resistances to VAN and ERY. Repeated transfer experiments were performed between *E. faecalis* FA2-2 and *E. faecalis* JH2SS. The vancomycin resistance was transferred at a frequency of about 10^−3^ per donor cell between these strains, and transconjugants obtained in each experiment also exhibited the same range of resistances as each of the donor strains. These results indicated that the conjugative plasmid encoded multiple-drug resistances. In Taiwan, besides avoparicin, macrolides and bacitracin have been used for food animals for a long time as growth promoters and keeping additives [40,41]. Bacitracin was limitedly used for humans as clinical treatment (external- and local-use only), and the usage amount for humans seemed to be not much compared with the usage for food animals [50]. These facts implied that the multiple-drug-resistant plasmids encoding bacitracin resistance gene cluster could be constructed, selected, and increased mainly in the surroundings (e.g., intestines) of food animals in Taiwan.

### 2.7. DNA Sequence Analysis of Pheromone-Responsive Conjugative Plasmid pTW9

**Figure 3:**
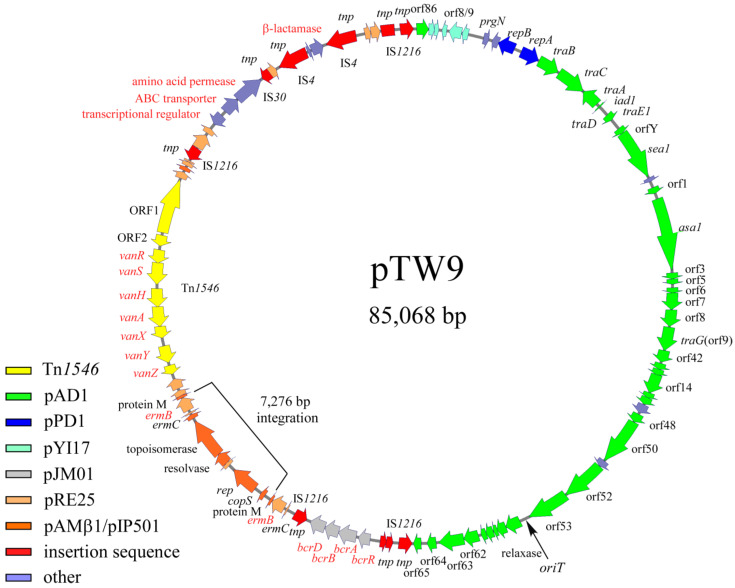
Genetic map of pTW9 (type A plasmid). The open arrows show the ORFs and the direction of transcription. Each color indicates significant homology with a reported plasmid or mobile element. Representative homologous genes are indicated on the ORFs (Table S2). The “A” residue next to the insertion point of IS*1216V* upstream of the orf86 homologue of pAD1 (orf4 homologue of pYI17) indicates the first nucleotide of pWT9 on the map, as shown in Supplementary Table S2. The 7276 bp integration region containing the replication gene and *erm* element (*erm*(C), *erm*(B), and protein M), which are homologous with pAMβ1 or the pRE25 plasmid, was not found in the pTW24 plasmid (type B plasmid, expected as 77,792 bp in size).

The complete nucleotide sequence of pTW9 was determined, and its molecular size was confirmed to be 85,068 bp (Figure 3, Table S2). The “A” residue next to the insertion point of IS*1216V* upstream of an ORF homologous to orf86 of pAD1 (a homologue of orf4 of pYI17) was chosen as the first nucleotide of pWT9 on the map, as shown in Supplementary Table S2 [30,51]. All ORFs listed in Table S2 are numbered in relation to this nucleotide. pTW9 encoded the drug resistance determinants for bacitracin (*bcrABDR*), macrolide (*erm*), and vancomycin(*vanA*), and the resistance genes lay between 44,905 bp and 69,606 bp of the pTW9 map.

The bacitracin resistance operon was composed of *bcrR*, *A*, *B*, and *D* located between 44,905 bp and 48,183 bp on the pTW9 map [43,50,52]. pTW9 encoded two *erm*(C), *erm*(B) determinants for macrolide resistance; one is located between 49,518 bp and 49,726 bp, and the determinant is located between 56,794 bp and 57,739 bp [53,54]. Since there were hairpin structures upstream of *erm* genes, the erythromycin resistances could be regulated and inducible [53]. The pTW9 plasmid carries the conjugative transposon Tn*1546* (10,851 bp) encoding VanA-type vancomycin resistance, and it is located between 58,830 bp and 69,680 bp in the counterclockwise orientation of the plasmid map [7]. The target site of Tn*1546* was five-base-pair “AAGCA” and was duplicated at the flanking regions. The target sequence was different from the reported hot spots [55]. The Tn*1546* of pTW9 contained eleven ORFs including ORF1, ORF2, *vanR*, *vanS*, *vanH*, *vanA*, *vanX*, *vanY,* and *vanZ* for the vancomycin resistance determinants, which correspond to the eleven equivalent ORFs in the prototype Tn*1546* in pIP816 [5,8]. The deduced amino acid sequence of the VanA determinants of pTW9 was identical with the exception of three amino acid substitutes in the *vanS* genes [38]. In addition, the predicted resistance genes to polyketide antibiotics (ORF86, 87, and 88), which were originally found in *Streptomyces* species, were also located between 73,006 bp and 74,818 bp [56]. Although the phenotypic analysis of the resistance was not performed in this study, the exporter genes might be functional and confer the resistance to polyketide antibiotics. Deduced ORF93 protein had partial homology with β-lactamase, but pTW9 did not confer ampicillin resistance (MIC <1 mg L^−1^). Several transposon-related or insertion sequence (IS)-related sequences such as transposase (or part of transposase) encoded on the mobile elements were found on the flanking regions of the resistance determinants on the plasmid. A part of IS*1216* elements, which is often found in enterococci, were flanking with the bacitracin-resistant determinants and also with the polyketide-resistant determinants, respectively. These determinants might be introduced into the plasmid as the composite mobile elements by transposition if not by the homologous recombination between the IS sequences.

The ORFs lying between ORF7 and ORF47, on an approximately 38.9 kbp region running from 3671 bp to 42,604 bp of the map, showed homology of between 80% and 100% amino acid identity with the genes or ORFs found in the pheromone-responding plasmids (pAD1, pPD1, and pCF10) [26,27,51]. ORF7 corresponds to *prgN* of pCF10, and ORF9 and ORF10 correspond to the plasmid maintenance genes for plasmid partition and replication of pPD1, respectively. Other ORFs lying between ORF11 and ORF47 correspond to the ORFs or genes found in pAD1. The region ORF11 to ORF15 was identical to *traB*, *traC*, *traA*, *iad1 (traD),* and *traE1*, respectively. *traB* encodes the pheromone-responding regulatory genes that shut down pheromone production or reduce endogenous pheromone levels, *traC* allows the cell surface receptor to take up exogenous pheromone, *traA* acts as the pheromone receptor and negative regulator for the downstream genes of *iad1*, *iad1* acts as the pheromone inhibitor, *traD* encodes a negative regulator for the pheromone response, and *traE1* is a key positive regulator for expressing ORFs downstream of the regulatory genes including *asa1* for aggregation substance [23]. The deduced ORF13, 14, and 15 proteins showed 100% amino acid identity with *traA* and *traE1* of pAD1, respectively. ORF16, 17, 19, and 20 downstream of the regulatory genes were highly homologous to *orfY*, *sea1*, *orf1*, and *asa1* of pAD1, respectively. ORFs downstream of ORF20 contained ORFs that correspond to orf3, 5, 6, 7, 8, 9/10, 10/11, 12 to 16, 48, 50 to 53, and 57 to 65 of pAD1 [51]. pTW9 does not encode ORFs corresponding ORF54 to 56, and 66 to 75 of pAD1.

pTW9 encodes several corresponding genes of pRE25, which is a large conjugative macrolide–lincosamide–streptogramin resistance enterococcal plasmid isolated from dry sausage in Europe [57]. Those genes seemed to be classified into two groups. One group was related to the erythromycin-resistant determinants (*erm*), and another was related to plasmid-maintenance genes including replication and a toxin–antitoxin system (Figure 3, Table S2).

### 2.8. Partial Nucleotide Sequence Analysis of Plasmid pTW24

DNA sequence analysis showed that pTW9 contained two *erm* elements (duplication of *erm*(C), *erm*(B), and protein M genes), and the DNA sequences of a 7276 bp region located between 50,602 bp and 57,877 bp of the map have a high degree of homology to the DNA sequence of the pAMβ1 plasmid (Table S2) [58]. The homologous region contains part of pAMβ1, including the replication genes and one *erm* element (Figure 3). The molecular size of pTW9 was about 7.3 kbp longer than that of pTW24. Partial DNA sequence analysis of the *ermB* region of plasmid pTW24 showed that pTW24 did not contain the 7276 bp fragment that is located in pTW9 and has high homology to pAMβ1 or pRE25 [57]. These data implied that pTW9 resulted from the integration of a 7276 bp fragment (pAMβ1-type erythromycin-resistant plasmid) into the pTW24 plasmid (the expected size was 77,792 bp) by homologous recombination between *erm* elements. Throughout the present study, any structural change in pTW9 (e.g., conversion to pTW24-like plasmid) was not observed, and pTW9 seemed to be relatively stable.

## 3. Materials and Methods

### 3.1. Bacterial Strains, Plasmids, and Media

The bacterial strains and plasmids used in this study are listed in Table 3. Forty VanA-type VRE isolates were obtained from human patients (named from TVH201 to TVH240), and thirty isolates were obtained from livestock sources (named from TVA101 to TVA130). Forty human isolates were obtained from forty individual patients in seven different hospitals (i.e., Hospitals A, B, C, D, E, F, and G) in Taiwan during a period of 29 months (from December 1999 to March 2002) [59,60]. Hospitals A, B, C, and D are located in northern, Hospital E is located in central, and Hospitals F and G are located in southern Taiwan. The numbers of isolates obtained from Hospitals A, B, C, D, E, F, and G were 10, 3, 1, 14, 2, 1, and 9, respectively. The specimens obtained from human patients were as follows: wound (15), blood (2), pus (4), urine (2), ascites (2), stool (1), and unknown origin (14). The specimens from livestock sources were thirteen chicken meats (CM) from different supermarkets and traditional markets in Taipei (from Oct. to Dec. 2001), seven pig wastes (PW) obtained from Taipei areas, and ten baby chicken feathers (CF) from separate chicken farms in Taiwan (in 2001). The thirty VRE isolates from livestock sources were chicken meat (from TVA101 to TVA113), pig waste (from TVA114 to TVA120), and chicken feather (from TVA121 to TVA130). The *E. faecalis* strains were grown in BBL^TM^ brain heart infusion broth and agar or Bacto^TM^ Todd–Hewitt broth (BD, Sparks, MD, USA) at 37 °C. N2GT broth (nutrient broth No. 2 (Oxoid Ltd., London, UK) supplemented with 0.2% glucose and 100 mM of Tris-HCl (pH 7.5)) was used in the sex pheromone experiments. *Escherichia coli* strains were grown in Luria–Bertani medium (Difco Laboratories, San Diego, CA, USA) at 37 °C.

The antibiotics used in this study were as follows: ampicillin (AMP), bacitracin (BAC), chloramphenicol (CHL), erythromycin (ERY), fusidic acid (FA), gentamicin (GEN), kanamycin (KAN), rifampin (RIF), spectinomycin (SPT), streptomycin (STR), tetracycline (TET), teicoplanin (TEC), and vancomycin (VAN). The following antibiotics were used at the indicated concentrations for the selection of *E. faecalis*: ERY, 12.5 mg L^−1^; STR, 250 mg L^−1^; SPT, 250 mg L^−1^; CHL, 20 mg L^−1^; RIF, 25 mg L^−1^; and FA, 25 mg L^−1^. The following antibiotics were used at the indicated concentrations for the selection of *E. coli*: AMP, 100 mg L^−1^, and CHL, 50 mg L^−1^. All antibiotics were obtained from Sigma Chemical Co. (Tokyo, Japan) or Wako Chemicals Co. (Osaka, Japan). 5-Bromo-4-chloro-3-indolyl-β-D-galactopyranoside (X-Gal) (Wako chemicals Co.) was used at 40 mg L^−1^.

### 3.2. Antimicrobial Susceptibility Testing

The MICs of the antibiotics were determined by the agar dilution method. Susceptibility testing and interpretation of the results were in compliance with standards recommended by the Clinical and Laboratory Standards Institutes (formerly NCCLS) [61]. *E. hirae* ATCC 9790 was used as a control strain.

### 3.3. Plasmid and DNA Methodology

Recombinant DNA techniques, analyses of plasmid DNA with restriction enzymes, and agarose gel electrophoresis were carried out using standard methods [62,63]. The introduction of plasmid DNA into bacterial cells was carried out by electrotransformation, as described previously [25]. Plasmid DNA was purified from *E. faecalis* by the alkali lysis method [62]. Restriction enzymes were purchased from New England Biolabs Japan, Ltd. (Tokyo, Japan) and Roche Diagnostics Japan (Tokyo, Japan). PCR was performed with a GeneAmp PCR System 9700 apparatus (Applied Biosystems, Foster, CA, USA). *Taq* DNA polymerase was obtained from Takara Bio Inc. (Shiga, Japan) or Toyobo Life Science (Osaka, Japan).

### 3.4. DNA Sequence Analysis

Prior to the sequence analysis, the physical maps of plasmids were determined as described in the previous reports to avoid the sequence errors [26,30]. Sequence analysis was performed with a Dye Terminator cycle sequencing kit (Applied Biosystems) and with a 377 DNA sequencer and 310 gene analyzer (ABI Prism). To determine the DNA sequences of plasmid pTW9 (type A, 85,068 bp) and pTW24 (type B, 77,792 bp), a shotgun cloning method and pUC18 cloning vector plasmid were used [63]. To determine the DNA sequences in the gap regions, PCR amplification was performed to obtain PCR products covering the gaps. The PCR products were sequenced directly using custom primers. Open reading frames (ORFs) were identified and analyzed initially with Genetyx (version 5.1) computer software. To search for putative genes, a homology search using the BLAST database was performed through the NCBI website (http://blast.ncbi.nlm.nih.gov/Blast.cgi (accessed on 16 March 2023)) [64].

### 3.5. DNA Sequence Analysis of the vanS Gene of the vanA Determinants

Twenty-two *E. faecalis* human isolates and 30 *E. faecalis* livestock isolates exhibiting high-level resistance to vancomycin (i.e., MIC ≧ 256 mg L^−1^) and sensitivity (or low-level resistance (i.e., MIC ≦ 32 mg L^−1^)) to teicoplanin were analyzed for the *vanS* gene polymorphism by sequencing the PCR products with *vanS*-specific primers [37,38].

### 3.6. Conjugation Experiments

Filter mating was performed as described previously with a donor/recipient ratio of 1:10 [65]. Overnight cultures of 0.05 mL of the donor and 0.45 mL of the recipient were each added to 4.5 mL of fresh THB broth, and the mixtures were incubated at 37 °C with gentle agitation. Broth mating was carried out for 4 h. Portions of the mixed cultures were then plated on solid media with appropriate selective antibiotics. In broth mating experiments, transconjugants were counted after 48 h of incubation at 37 °C. Transfer frequencies were expressed as the number of transconjugants per donor cell (at the end of mating). *E. faecium* BM4105RF and *E. faecalis* FA2-2 were used as recipient strains in mating experiments with VRE isolates from human and livestock sources (Table 3).

### 3.7. Pheromone Response (Clumping) Assay

Pheromone response assays were performed as described previously [66]. The synthetic enterococci pheromones cAD1, cCF10, cPD1, cOB1, and cAM373 were prepared by Sawaday Technology Co., Ltd. (Tokyo, Japan) [10,23]. Synthetic pheromones (final concentration, 100 ng/mL) in N2GT broth were used in the experiments. The *E. faecalis* FA2-2 transconjugants were examined for pheromone response and were exposed for 2 h to either FA2-2 culture filtrate (i.e., pheromone) or the synthetic pheromones cAD1, cPD1, cCF10, cOB1, or cAM373 for the pheromone-responsive plasmids pAD1, pPD1, pCF10, pOB1, and pAM373, respectively, to induce the aggregation [10,23,24].

### 3.8. Pulsed-Field Gel Electrophoresis (PFGE)

Lysis of cells in agarose plugs was performed according to the standard protocols [62], except that the cells were treated with lysozyme at a concentration of 20 mg/mL. The reaction mixture for SmaI digestion of whole chromosomal DNA was incubated at 25 °C overnight. PFGE was carried out in a 1% agarose gel with 0.5% Tris-borate-EDTA buffer, and the following settings were applied: 1 to 23 s, 6 V/cm, 15 °C and 22 h (with the CHEF Mapper system (Bio-Rad Laboratories, Richmond, CA, USA)). The guidelines proposed by Tenover et al. were basically used for the interpretation of PFGE results in this study [67]. With these guidelines, strains that differed by between one and six bands were considered to be related clones, and the banding pattern difference of three fragments could have occurred due to a single genetic event.

### 3.9. Multi-Locus Sequence Typing (MLST) Analyses

Internal fragments of the *aroE*, *gdh*, *gki*, *gyd*, *pstS*, *xpt,* and *ygiL* genes, which are the seven housekeeping genes of *E. faecalis*, were amplified by PCR from chromosomal DNA using the primer pairs described in the MLST database (https://pubmlst.org/organisms/enterococcus-faecalis (accessed on 16 March 2023)). The seven housekeeping genes were sequenced, and the allele numbers were determined according to the database. New ST numbers (i.e., ST263, ST264, ST265, and ST266) in this study were determined by the MLST database administrators and registered on the database.

### 3.10. Nucleotide Sequence Accession Number

The nucleotide sequence data reported in this article are available from the DDBJ, EMBL, and GenBank nucleotide sequence databases under accession number AB563188.

## 4. Conclusions

In this study, we focused on the VanB-like phenotypic VanA-genotype *E. faecalis* isolated in the early 2000s to elucidate the relationship between the livestock VRE and human VRE in Taiwan. Our data showed evidence for the dissemination of VAN^r^/ERY^r^/BAC^r^ encoded on the similar highly conjugative plasmids in both livestock VRE and human VRE. We also revealed the transmissions of VRE strains between livestock and humans, and the VRE had been most likely transmitted from livestock to humans in Taiwan around 2000. To our knowledge, this is the first report of the detection of multiple indistinguishable (genetically identical) VRE strains in both livestock and humans (inpatients).

To date, most VRE isolates are found in *E. faecium*. However, compared with Europe and the US, VanA-type *E. faecalis* strains have been relatively isolated in the East Asian and Oceanic countries, although the reason was unknown. Our national surveillance data showed that about 48% of VRE clinical isolates have been *E. faecalis* strains in Japan (unpublished data). As shown here, in the early 2000s in Taiwan, all of the VanA-type VRE isolates were obtained from livestock, and more than half (57.5%) of human clinical isolates have also been *E. faecalis*. Almost all of the *E. faecalis* strains showed high-level resistance to vancomycin and sensitivity to teicoplanin (VanB-like phenotype) and had three amino acids substitutions in the *vanS* gene of the *vanA* determinant which are frequently found in the VanA-type VRE isolated from chicken imported to Japan from Thailand, and also found in poultry food samples in other East Asian countries and Europe. The *vanA* determinant was encoded on Tn*1546,* and the Tn*1546* was located on the *E. faecalis* pheromone-responsive conjugative plasmids. Two closely related plasmid types that responded to pheromone cAD1 were identified in both human and livestock isolates. Many of the VRE isolated from patients and livestock sources showed variations in PFGE of the SmaI fragments of the chromosomal DNA. Two strains, which were isolated from patients and livestock, respectively, showed indistinguishable PFGE profiles and harbored an identical pheromone-responsive plasmid, providing definite evidence of the transmission of VRE between livestock and humans. The complete nucleotide sequence analysis of the representative pheromone-responsive plasmid pTW9 (85,068 bp) showed that the plasmid encodes multiple-drug resistances, including vancomycin, erythromycin, and bacitracin, and the transfer-related genes of pAD1-type plasmid. Our results showed that the corresponding pheromone-responsive plasmids which encoded multiple-drug resistances, including *vanA* resistance, have been widely spread in both human and livestock *E. faecalis* isolates in Taiwan in the early 2000s. Before the 20th century in Taiwan, live food animals were frequently traded in the traditional markets, and people sacrificed and cooked them at home. The animal foods may have been one of the transmission routes of VRE, and the drug resistances could be transmitted from livestock to humans, although the possibility of the existence of a common reservoir for VRE could not be excluded in this study. It may be possible that Taiwan’s traditional dietary habits and limited regional characteristics as an island nation contributed to the transmission and spread of VRE among humans and livestock within Taiwan. We also speculated that the dissemination of multiple-drug-resistant pheromone-responsive plasmids may have been one of the reasons why VRE strains have been relatively found in *E. faecalis* in the East Asian and Oceanic countries. Our data indicated that, even after banning the use of avoparcin, there was the possibility of spreading of multidrug-resistant pheromone-responsive plasmids (VCM^r^, ERY^r^, BAC^r^) among *E. faecalis*, resulting in the increase in VRE in livestock under the selective pressure of the other antibiotics such as ERY and BAC. Therefore, it should be necessary to continue to carefully monitor the use of antibiotics in agricultural settings and the status of multidrug-resistant enterococci in livestock in these countries.

## Figures and Tables

**Table 1:** Antimicrobial Resistance in Vancomycin-resistant *E. faecalis*.

Drug	No. of Drug-Resistant Isolates (%) *^a^*	
	From Human	From Livestock
	*n* = 22 *^b^*	*n* = 30 *^b^*
AMP	0 (0)	0 (0)
BAC	21 (95.5)	29 (96.7)
CHL	18 (81.8)	25 (83.3)
CIP	6 (27.3)	5 (16.7)
ERY	22 (100)	30 (100)
GEM	8 (36.4)	8 (26.7)
KAN	21 (95.5)	25 (83.3)
SPT	14 (63.6)	13 (43.3)
STR	14 (63.6)	22 (73.3)
TEC	1 (4.5)	3 (10)
TET	22 (100)	30 (100)
VAN	22 (100)	30 (100)

*^a^* The numbers in parentheses indicate the percentages of resistant isolates. The drug resistance levels (MICs) of ampicillin(AMP), bacitracin(BAC), chloramphenicol(CHL), ciprofloxacin(CIP), erythromycin(ERY), gentamicin(GEM), kanamycin(KAN), spectinomycin(SPT), streptomycin(STR), teicoplanin(TEC), tetracycline(TET), and vancomycin(VAN) were equal to or greater than 16, 256, 32, 4, 8, 512, 512, 1024, 512, 32, 16, and 32 mg/L, respectively. *^b^* n, number of strains tested.

**Table 2:** VanA-type *E. faecalis* Strains from Human and Livestock and Their Characteristics.

Strain	*van* Gene (Phenotype *^a^*)	Hospital (Location)	Specimen	MLST *^b^* (Closely-Related ST) *^c^*	Plasmid Content *^d^*	Response to Pheromone *^e^*	Drug Resistance Level (MIC, mg/L) *^f^*											
							VAN	TEC	AMP	CIP	ERY	GEN	KAN	STR	CHL	TET	SPC	BAC
TVH209	*vanA* (VanB)	G (southern)	blood (human)	ST263 (ST33, 59)	pTW9	cAD1	512	8	<1	32	>1024	<16	>1024	>1024	128	>128	64	>256
TVA126	*vanA* (VanB)		chicken feather	ST263 (ST33, 59)	pTW9-like	cAD1	512	8	<1	64	>1024	<16	>1024	>1024	64	128	64	>256
TVH208	*vanA* (VanB)	G (southern)	wound (human)	ST264 (ST86)	unknown type	unknown type	256	8	<1	<4	>1024	>1024	>1024	512	64	128	1024	64
TVH217	*vanA* (VanB)	D (northern)	abscess (human)	ST264 (ST86)	unknown type	cAD1	512	8	<1	<4	>1024	>1024	>1024	512	8	128	1024	>256
TVA122	*vanA* (VanB)		chicken feather	ST264 (ST86)	pTW24-like	cAD1	512	>16	<1	<4	>1024	<16	>1024	1024	8	128	>1024	>256
TVH222	*vanA* (VanB)	D (northern)	wound (human)	ST265 (ST16)	unknown type	unknown type	256	2	<1	<4	>1024	32	>1024	512	8	128	>1024	>256
TVA117	*vanA* (VanB)		pig waste	ST265 (ST16)	non-conjugative		512	16	<1	<4	>1024	128	>1024	512	8	128	>1024	>256
TVA113	*vanA* (VanB)		chicken meat	ST266 (ST93)	pTW24-like	cAD1	512	8	<1	<4	>1024	<16	32	32	64	128	64	>256
TVA118	*vanA* (VanB)		pig waste	ST266 (ST93)	pTW24-like	cAD1	512	8	<1	<4	>1024	<16	>1024	>1024	64	128	64	>256
FA2-2							<1	<1	<1	<4	<1	<16/(8)	64	64	8	<4	64	16

*^a^* The VanB phenotype was caused by three amino acid substitutions in the VanS encoded on the *vanA*-type transposon Tn*1546* [38]. *^b^* MLST were determined from the sequence data of seven house-keeping gene alleles including *aroE*, *gdh*, *gki*, *gyd*, *pstS*, *xpt* and *ygiL* (https://pubmlst.org/organisms/enterococcus-faecalis, accessed on 16 March 2023). *^c^* Reported closely-related MLST types are shown (single- or double- locus variants). ST33 and ST59 strains are isolated from human and animal (poultry) [39,44,45]. ST16 strains form the clonal complex CC16 and are isolated from swine, poultry and human [44,46,47]. ST93 strain originally isolated from human feces [44]. *^d^* Vancomycin resistance pheromone-responsive highly-conjugative plasmid; unknown type, classified into unknown type based on the EcoRI-digested plasmid DNA profiles. *^e^* The specific synthetic pheromone induced the cell aggregations; unknown type means an unknown pheromone other than cAD1, cCF10, cPD1, cAM373, and cOB1 induced the aggregation. *^f^* Drug abbreviations: VAN, vancomycin; TEC, teicoplanin; AMP, ampicillin; CIP, ciprofloxacin; ERY, erythromycin; GEN, gentamicin; KAN, kanamycin; STR, streptomycin; CHL, chloramphenicol; TET, tetracycline; SPT, spectinomycin; BAC, bacitracin.

**Table 3:** Bacterial Strains and Plasmids used in this Study.

	Relevant Features and Origins (Specimens)	Reference
Strains:		
VanA-type VRE		
TVH201 to TVH240	forty isolates from individual patients of seven hospitals (hospital A, B, C, D, E, F and G) in Taiwan	This study, [59,60]
	(TVH201 to TVH209; hospital G, TVH210 and TVH211; hospital A, TVH212 to TVH225; hospital D,	
	TVH226; hospital B, TVH227; hospital C, TVH228, hospital F, TVH229 to TVH236; hospital A,	
	TVH237 and TVH239; hospital E, TVH238 and TVH240; hospital B)	
TVA101 to TVA130	thirty isolates from separate livestock in Taiwan	This study, [59,60]
	(TVA101 to TVA113; chicken meat [CM], TVA114 to TVA120; pig waste [PW],	
	TVA121 to TVA130; chicken feather [CF])	
FA2-2	*rif*, *fus,* derivative of JH2	[49]
JH2SS	*spc*, *str,* derivative of JH2	[54]
BM4105RF	*rif*, *fus*, derivative of plasmid-free *E. faecium* BM4105	[11]
BM4105SS	*spc*, *str*,derivative of plasmid-free *E. faecium* BM4105	[11]
ATCC9790	penicillin susceptible, PBP5 low producer	
DH5a	*endA1 recA1 gyrA96 thi-1 hsdR17 supE44 relA1 ∆(argE-lacZYA) U169*	Bethesda Research Laboratories
pUC18	*E. coli* cloning vector, Amp^r^	Nippon gene
pTW9	pheromone (cAD1) responsive plasmid isolated from TVH209, 85.1 kbp, VAN^r^, ERY^r^, BAC^r^	This study
	predicted derivative of pTW24-like plasmid by integration of a 7.3 kbp ERY^r^ plasmid	
	designated as type A plasmid in this study	
pTW24	pheromone (cAD1) responsive plasmid isolated from TVH224, 77.8 kbp, VAN^r^, ERY^r^, BAC^r^	This study
	designated as type B plasmid in this study	

## Data Availability

Data is contained within the article or Supplementary Material.

## Supplementary Materials

The following supporting information can be downloaded at: https://www.mdpi.com/article/10.3390/antibiotics12121668/s1, Figure S1:Pulsed-field gel electrophoresis (PFGE) of SmaI-digested total DNAs of human (patients) VanA-genotype VRE isolates showing VanB-like phenotype in Taiwan; Figure S2: Pulsed-field gel electrophoresis (PFGE) of SmaI-digested total DNAs of livestock VanA-genotype VRE isolates showing VanB-like phenotype in Taiwan; Table S1: Drug susceptibility (MIC values: mg/L) of VanA-genotype/VanB-like phenotype *E. faecalis* strains isolated from human (22 patients) and livestock (30 Samples) in Taiwan; Table S2: Open reading frames of pTW9 (85,068 bp; VAN^r^, ERY^r^, BAC^r^).
