# Supplementary material for: High Incidence of Multiple-Drug-Resistant Pheromone-Responsive Plasmids and Transmissions of VanA-Type Vancomycin-Resistant Enterococcus faecalis between Livestock and Humans in Taiwan
Source: Antibiotics (Basel). 2023 Nov 27;12(12):1668. doi: 10.3390/antibiotics12121668 (PMC10740520; doi:10.3390/antibiotics12121668)
Supplement: Supplementary file 1 [file antibiotics-12-01668-s001.zip › antibiotics-2681737-supplementary.pdf]

Figure S1.

Pulsed-field gel electrophoresis (PFGE) of SmaI-digested total DNAs of human (patients) VanA-genotype VRE isolates showing VanB-like phenotype in Taiwan

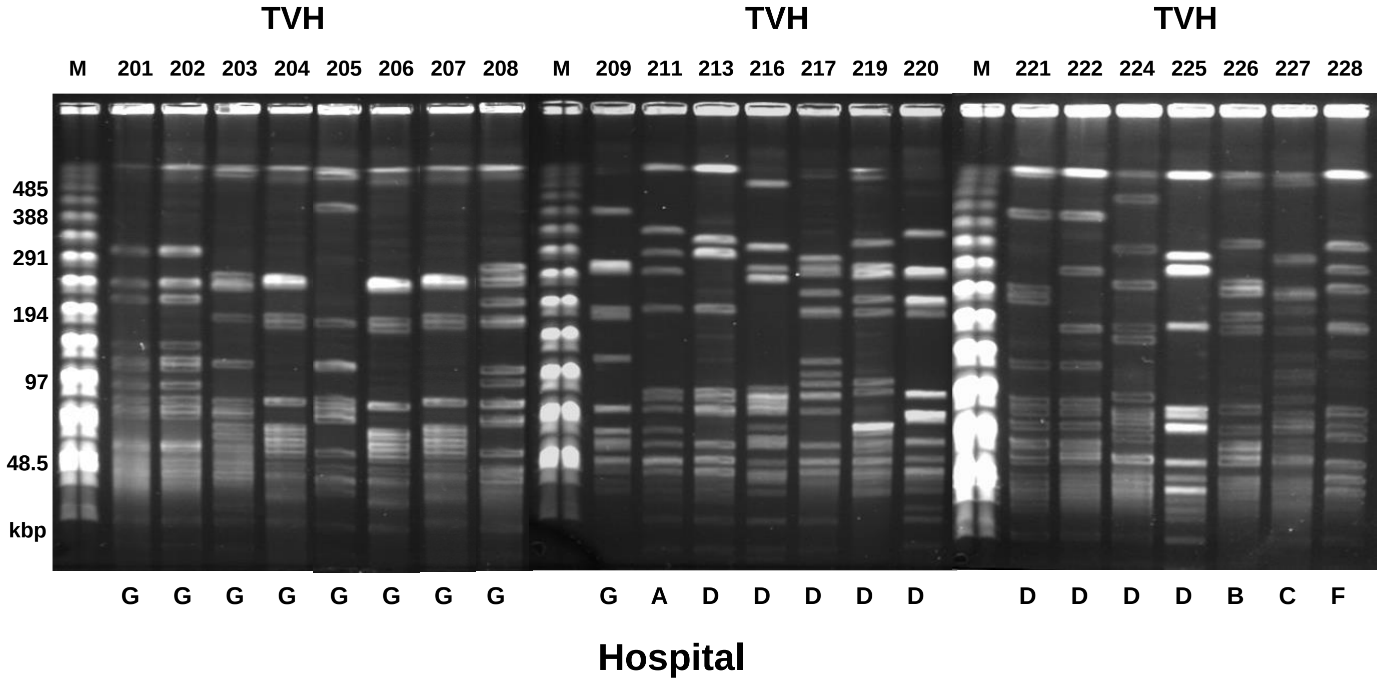

M, Lambda ladder PFGE marker (New England Biolabs, Beverly, MA)

Figure S2.

Pulsed-field gel electrophoresis (PFGE) of SmaI-digested total DNAs of livestock VanA-genotype VRE isolates showing VanB-like phenotype in Taiwan

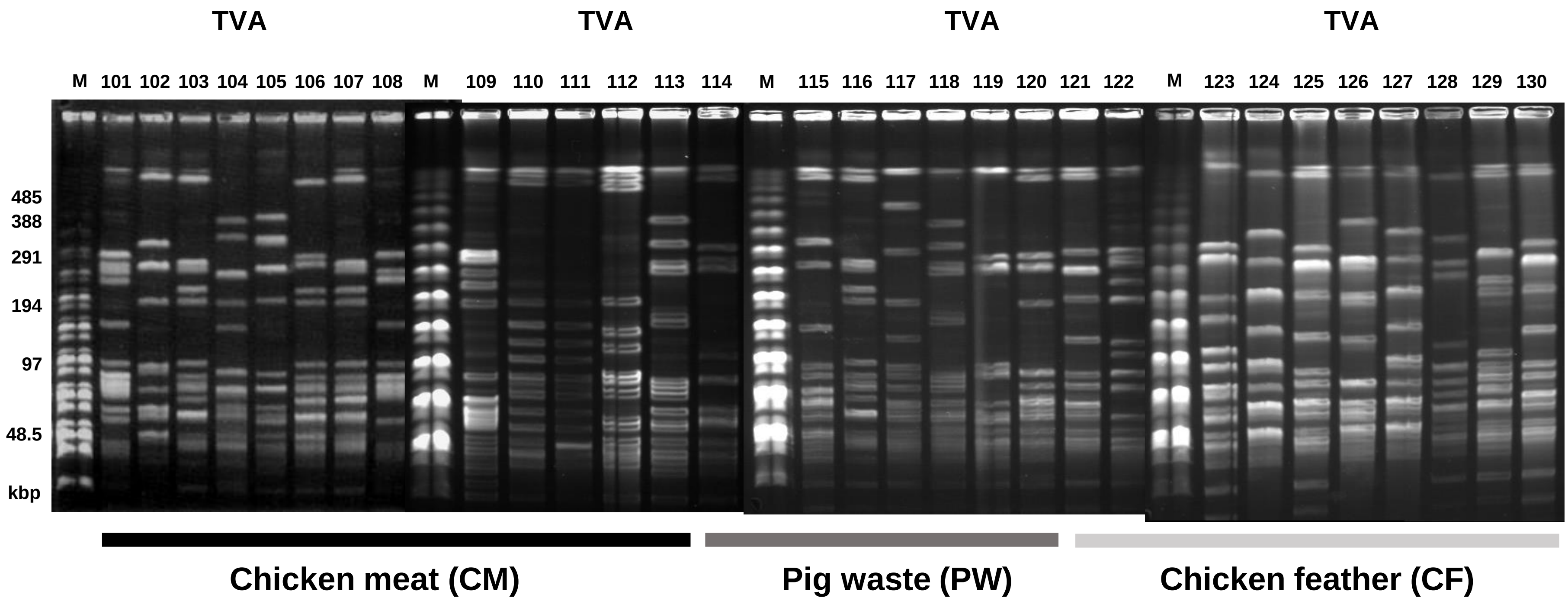

M, Lambda ladder PFGE marker (New England Biolabs, Beverly, MA)

Table S1. Drug Susceptibility (MIC Values: mg/L) of VanA-Genotype/VanB-like Phenotype *E. faecalis* Strains isolated from Human (22 Patients) and Livestock (30 Samples) in Taiwan.

Strain	Hospital (Location in Taiwan)	Specimen	Species	Genotype (Phenotype)	MLST	VAN^r plasmid (Type)	Pheromone	AMP	BAC	CHL	CIP	ERY	GEM	KAN	SPT	STR	TEC	TET	VAN
TVH201	G (southern)	ascites	*E. faecalis*	*vanA* (VanB)			cAD1	<1	>256	32	<4	>1,024	256	>1,024	>1,024	512	4	128	512
TVH202	G (southern)	wound	*E. faecalis*	*vanA* (VanB)			cAD1	<1	>256	32	<4	>1,024	256	>1,024	>1,024	512	4	128	512
TVH203	G (southern)	wound	*E. faecalis*	*vanA* (VanB)			unknown	<1	>256	32	32	>1,024	>1,024	>1,024	64	>1,024	4	128	256
TVH204	G (southern)	urine	*E. faecalis*	*vanA* (VanB)			unknown	<1	>256	128	<4	>1,024	<16	>1,024	1024	>1,024	<0.25	128	256
TVH205	G (southern)	pus	*E. faecalis*	*vanA* (VanB)			cAD1	<1	>256	64	<4	>1,024	1,024	>1,024	>1,024	>1,024	4	>128	512
TVH206	G (southern)	wound	*E. faecalis*	*vanA* (VanB)			unknown	<1	>256	128	<4	>1,024	<16	>1,024	1,024	>1,024	1	>128	512
TVH207	G (southern)	wound	*E. faecalis*	*vanA* (VanB)			unknown	<1	>256	128	<4	>1,024	<16	>1,024	1,024	>1,024	1	>128	256
TVH208	G (southern)	wound	*E. faecalis*	*vanA* (VanB)	ST264		unknown	<1	64	64	<4	>1,024	>1,024	>1,024	1,024	512	8	128	256
TVH209	G (southern)	blood	*E. faecalis*	*vanA* (VanB)	ST263	pTW9 (Type A)	cAD1	<1	>256	128	32	>1,024	<16	>1,024	64	>1,024	8	>128	512
TVH211	A (northern)	wound	*E. faecalis*	*vanA* (VanB)			cOB1	<1	>256	64	32	>1,024	>1,024	>1,024	>1,024	>1,024	4	128	512
TVH213	D (northern)	wound	*E. faecalis*	*vanA* (VanB)			cOB1	<1	>256	64	64	>1,024	>1,024	>1,024	>1,024	>1,024	8	128	512
TVH216	D (northern)	wound	*E. faecalis*	*vanA* (VanB)		pTW9-like (Type A)	cAD1	<1	>256	8	<4	>1,024	<16	1,024	64	256	4	128	512
TVH217	D (northern)	pus (abscess)	*E. faecalis*	*vanA* (VanB)	ST264		cAD1	<1	>256	8	<4	>1,024	>1,024	>1,024	1,024	512	8	128	512
TVH219	D (northern)	wound	*E. faecalis*	*vanA* (VanB)			cOB1	<1	>256	64	<4	>1,024	<16	>1,024	1,024	>1,024	>16	128	512
TVH220	D (northern)	ascites	*E. faecalis*	*vanA* (VanB)			cAD1	<1	>256	8	<4	>1,024	<16	128	64	32	8	128	512
TVH221	D (northern)	wound	*E. faecalis*	*vanA* (VanB)			cOB1	<1	>256	32	<4	>1,024	>1,024	>1,024	>1,024	>1,024	16	128	512
TVH222	D (northern)	wound	*E. faecalis*	*vanA* (VanB)	ST265		unknown	<1	>256	8	<4	>1,024	32	>1,024	>1,024	512	2	128	256
TVH224	D (northern)	pus (ear)	*E. faecalis*	*vanA* (VanB)		pTW24 (Type B)	cAD1	<1	>256	32	<4	>1,024	1,024	>1,024	64	>1,024	8	128	512
TVH225	D (northern)	urine	*E. faecalis*	*vanA* (VanB)		pTW9-like (Type A)	cAD1	<1	>256	64	64	>1,024	<16	>1,024	64	>1,024	8	128	512
TVH226	B (northern)	pus (drain)	*E. faecalis*	*vanA* (VanB)			cAD1	<1	>256	128	<4	>1,024	<16	>1,024	1,024	256	8	128	512
TVH227	C (northern)	unknown	*E. faecalis*	*vanA* (VanB)		pTW24-like (Type B)	cAD1	<1	>256	64	<4	>1,024	<16	>1,024	64	1,024	4	128	512
TVH228	F (southern)	unknown	*E. faecalis*	*vanA* (VanB)			cOB1	<1	>256	64	64	>1,024	32	>1,024	512	>1,024	16	128	512

Strain (Sample #)	Livestock source	Species	Genotype (Phenotype)	MLST	Plasmid (Vm^r)	Pheromone	AMP	BAC	CHL	CIP	ERY	GEM	KAN	SPT	STR	TEC	TET	VAN
TVA101 (101)	chicken meat [CM]	*E. faecalis*	*vanA* (VanB)			unknown type	<1	>256	64	<4	>1,024	128	>1,024	>1,024	>1,024	4	128	512
TVA102 (102)	chicken meat [CM]	*E. faecalis*	*vanA* (VanB)		pTW9-like (Type A)	cAD1	<1	256	32	<4	>1,024	<16	64	64	512	4	128	512
TVA103 (103)	chicken meat [CM]	*E. faecalis*	*vanA* (VanB)			cOB1	<1	>256	32	<4	>1,024	512	>1,024	>1,024	>1,024	16	128	512
TVA104 (104)	chicken meat [CM]	*E. faecalis*	*vanA* (VanB)		pTW24-like (Type B)	cAD1	<1	>256	8	<4	>1,024	<16	1,024	64	64	4	128	512
TVA105 (105)	chicken meat [CM]	*E. faecalis*	*vanA* (VanB)			unknown type	<1	64	64	<4	>1,024	<16	64	64	>1,024	8	128	512
TVA106 (106)	chicken meat [CM]	*E. faecalis*	*vanA* (VanB)		non-conjugative		<1	>256	32	<4	>1,024	<16	>1,024	64	1,024	16	128	512
TVA107 (107)	chicken meat [CM]	*E. faecalis*	*vanA* (VanB)		non-conjugative		<1	>256	64	<4	>1,024	256	>1,024	>1,024	>1,024	>16	128	512
TVA108 (108)	chicken meat [CM]	*E. faecalis*	*vanA* (VanB)		non-conjugative		<1	>256	64	<4	>1,024	256	>1,024	1,024	>1,024	16	128	512
TVA109 (109)	chicken meat [CM]	*E. faecalis*	*vanA* (VanB)		non-conjugative		<1	>256	64	<4	>1,024	<16	>1,024	>1,024	>1,024	8	128	512
TVA110 (110)	chicken meat [CM]	*E. faecalis*	*vanA* (VanB)		non-conjugative		<1	>256	32	<4	>1,024	1,024	>1,024	>1,024	>1,024	16	128	512
TVA111 (111)	chicken meat [CM]	*E. faecalis*	*vanA* (VanB)			cOB1	<1	>256	32	<4	>1,024	512	>1,024	>1,024	>1,024	16	128	512
TVA112 (112)	chicken meat [CM]	*E. faecalis*	*vanA* (VanB)			cAD1	<1	>256	8	<4	>1,024	512	>1,024	>1,024	1,024	8	128	512
TVA113 (118)	chicken meat [CM]	*E. faecalis*	*vanA* (VanB)	ST266	pTW24-like (Type B)	cAD1	<1	>256	64	<4	>1,024	<16	32	64	32	8	128	512
TVA114 (119)	pig waste [PW]	*E. faecalis*	*vanA* (VanB)			unknown type	<1	>256	64	<4	>1,024	<16	>1,024	1,024	>1,024	8	128	512
TVA115 (121)	pig waste [PW]	*E. faecalis*	*vanA* (VanB)		non-conjugative		<1	>256	64	<4	>1,024	<16	32	64	>1,024	16	128	512
TVA116 (122)	pig waste [PW]	*E. faecalis*	*vanA* (VanB)		non-conjugative		<1	>256	64	<4	>1,024	512	>1,024	>1,024	>1,024	>16	128	512
TVA117 (123)	pig waste [PW]	*E. faecalis*	*vanA* (VanB)	ST265	non-conjugative		<1	>256	8	<4	>1,024	128	>1,024	>1,024	512	16	128	512
TVA118 (124)	pig waste [PW]	*E. faecalis*	*vanA* (VanB)	ST266	pTW24-like (Type B)	cAD1	<1	>256	64	<4	>1,024	<16	>1,024	64	>1,024	8	128	512
TVA119 (125)	pig waste [PW]	*E. faecalis*	*vanA* (VanB)		pTW24-like (Type B)	cAD1	<1	>256	64	<4	>1,024	<16	>1,024	64	>1,024	16	128	512
TVA120 (126)	pig waste [PW]	*E. faecalis*	*vanA* (VanB)			cAD1	<1	>256	64	64	>1,024	>1,024	>1,024	64	>1,024	16	128	512
TVA121 (152)	chicken feather [CF]	*E. faecalis*	*vanA* (VanB)		pTW9-like (Type A)	cAD1	<1	>256	64	32	>1,024	>1,024	>1,024	64	64	16	128	512
TVA122 (153)	chicken feather [CF]	*E. faecalis*	*vanA* (VanB)	ST264	pTW24-like (Type B)	cAD1	<1	>256	8	<4	>1,024	<16	>1,024	>1,024	1,024	>16	128	512
TVA123 (154)	chicken feather [CF]	*E. faecalis*	*vanA* (VanB)		non-conjugative		<1	>256	16	<4	>1,024	<16	>1,024	64	>1,024	4	128	512
TVA124 (158)	chicken feather [CF]	*E. faecalis*	*vanA* (VanB)		pTW9-like (Type A)	cAD1	<1	>256	64	<4	>1,024	<16	512	64	1,024	8	128	512
TVA125 (160)	chicken feather [CF]	*E. faecalis*	*vanA* (VanB)			cOB1	<1	>256	64	64	>1,024	<16	1,024	>1,024	64	8	128	512
TVA126 (162)	chicken feather [CF]	*E. faecalis*	*vanA* (VanB)	ST263	pTW9-like (Type A)	cAD1	<1	>256	64	64	>1,024	<16	>1,024	64	>1,024	8	128	512
TVA127 (163)	chicken feather [CF]	*E. faecalis*	*vanA* (VanB)		pTW9-like (Type A)	cAD1	<1	>256	64	<4	>1,024	<16	64	64	1,024	4	128	512
TVA128 (164)	chicken feather [CF]	*E. faecalis*	*vanA* (VanB)				<1	>256	64	<4	>1,024	<16	>1,024	64	64	16	128	512
TVA129 (167)	chicken feather [CF]	*E. faecalis*	*vanA* (VanB)		pTW24-like (Type B)	cAD1	<1	>256	32	<4	>1,024	1,024	>1,024	64	1,024	8	128	256
TVA130 (169)	chicken feather [CF]	*E. faecalis*	*vanA* (VanB)			unknown type	<1	>256	64	64	>1,024	<16	>1,024	64	64	2	128	512

The numbers indicate the MIC values (mg/L) of antibiotics. The drug resistance levels 'R' of ampicillin (AMP), bacitracin (BAC), chloramphenicol (CHL), ciprofloxacin (CIP), erythromycin (ERY), gentamicin (GEM), kanamycin (KAN), spectinomycin (SPT), streptomycin (STR), teicoplanin (TEC), tetracycline (TET), and vancomycin (VAN) were equal to or greater than 16, 256, 32, 4, 8, 512, 512, 1024, 512, 32, 16, and 32 (mg/L), respectively [61].

Table S2. Open Reading Frames of pTW9 (85,068 bp; VAN^{r}, ERY^{r}, BAC^{r})

ORF[a]	5′	3′	Dir.	bp	a.a.	G+C (%)	Homology	Identity /similarity(%)	Organism	Function
1	118	738	F	621	206	33.1	ORF86 (orf4)	97/99	pAD1(pYI17)	DNA invertase
2	755	1039	F	285	94	30.9	ORF5	100/100	pYI17	
3	1041	1274	F	234	77	30.8	ORF6	100/100	pYI17	
4	1434	1688	R	255	84	25.9	ORF7	100/100	pYI17	
5	1805	2473	R	669	222	28.2	ORF8/ORF9	100/100	pYI17	abortive infection protein, putative protease
6	2509	2826	R	318	105	33.2	ORF10	100/100	pYI17	
7	3671	3967	F	297	97	34.6	*prgN*	96/98	pCF10	plasmid replication control
8	4126	4467	R	342	113	32.0	pXO2-40	31/43	*Bacillus anthracis* pXO2	replication-associated protein
9	4471	5373	R	903	300	30.7	*repB*	100/100	pPD1	parA family protein
10	5769	6776	F	1008	335	29.9	*repA*	93/96	pPD1	replication protein
11	6820	7986	F	1167	388	29.5	*traB*	99/99	pAD1	pheromone shutdown protein: traB family
12	8051	9643	F	1593	530	33.1	*traC*	99/99	pAD1	extracellular solute binding protein
13	9644	10603	R	960	319	26.8	*traA*	100/100	pAD1	pheromone-binding protein / negative regulator
14	10786	10854	F	69	22	27.7	*iad1*	100/100	pAD1	iAD1precouser (MSKRAMKKIIPLIT/LFVVTLVG)
15	11422	11778	F	357	118	34.4	*traE1*	100/100	pAD1	positive regulator
16	12360	12665	F	306	101	36.1	*orfY*	99/100	pAD1	
17	12676	15348	F	2673	890	38.3	*sea1*	96/98	pAD1	surface exclusion
18	15420	15605	F	186	61	31.3	EF0051	97/98	V583	
19	15945	16280	F	336	111	33.7	orf1	100/100	pAD1	
20	16587	20477	F	3891	1296	38.6	*asa1*	99/99	pAD1	aggregation substance
21	20572	20892	F	321	106	32.9	orf3	100/100	pAD1	
22	20914	21174	F	261	86	31.0	orf5	98/100	pAD1	
23	21368	21628	F	261	86	32.4	orf6	80/80	pAD1	signal seq
24	21639	22517	F	879	292	33.2	orf7	97/97	pAD1	transmemb protein
25	22537	23397	F	861	286	42.7	orf8	48/48	pAD1	cell wall anchoring signal
26	23423	24694	F	1272	423	40.4	*traG* (orf9)	99/99	pAD1	signal seq, transmemb protein
27	24697	25314	F	618	205	35.4	orf42	99/99	pAD1	signal seq
28	25382	25714	F	333	110	36.4	orf12	98/99	pAD1	signal seq
29	25714	26028	F	315	104	40.8	orf13	99/100	pAD1	
30	26021	27055	F	1035	344	36.0	EFA0037	99/99	pTEF1	signal seq
31	27060	27320	F	261	86	36.7	EFA0036	100/100	pTEF1	signal seq, transmemb protein
32	27320	27709	F	390	129	33.2	orf16	100/100	pAD1	transmemb protein
33	27741	28223	F	483	160	31.8	EF0498	99/100	V583	
34	28316	28774	F	459	152	38.6	orf48	100/100	pAD1	putative ssb protein
35	28807	31371	F	2565	854	37.3	orf50	99/99	pAD1	ATP binding domain
36	31382	31723	F	342	113	29.8	EFA0032	99/100	V583	lipoprotein
37	31741	34086	F	2346	781	36.7	orf52	97/98	pAD1	transmemb protein
38	34088	36331	F	2244	747	35.8	orf53	98/99	pAD1	signal seq, transmemb , ATP binding
39	36854	37639	F	786	261	30.2	orf57	99/99	pAD1	relaxase
40	37645	38133	F	489	162	30.8	orf58	99/99	pAD1	
41	38126	38329	F	204	67	41.6	orf59	100/100	pAD1	
42	38359	38733	F	375	124	38.2	orf60	99/99	pAD1	
43	38740	39006	F	267	88	37.9	orf61	99/99	pAD1	
44	39168	39944	F	777	258	36.2	orf62	99/99	pAD1	
45	39941	41275	F	1335	444	37.2	orf63	99/100	pAD1	
46	41415	41891	F	477	158	42.2	pTEF0019 (orf64)	100/100(91/91)	pTEF1(pAD1)	
47	42194	42604	F	411	136	41.2	orf65	100/100	pAD1	
48	42664	43350	R	687	228	37.6	transposase	100/100	IS*1216*	transposase
49	43669	44016	R	348	116	37.7	truncated transposase	88/93	IS*1216V*	truncated transposase
50	44017	44355	R	339	112	37.7	truncated transposase	88/93	IS*1216V*	truncated transposase
51	44905	45519	F	615	204	38.4	BcrR	97/98	pJM01[b]	DNA-binding protein
52	45646	46611	F	966	321	40.8	BcrA	100/100	pJM01[b]	ATP-binding domain of ABC transporter
53	46589	47353	F	765	254	39.4	BcrB	99/100	pJM01[b]	membrane-bound permease of ABC transporter
54	47353	48183	F	831	276	40.1	BcrD	100/100	pJM01[b]	bacitracin resistance protein[d]
55	48381	49067	R	687	228	37.5	transposase	90/95	IS*1216*	transposase
56	49518	49601	F	84	27	29.5	orf13, *erm* (C)	100/100	pRE25[c]	MSL leader peptide
57	49726	50463	F	738	245	33.2	orf14, *erm* (B)	100/100	pRE25[c]	erythromycin resistance protein
58	50468	50599	F	132	43	38.8	orf15 (protein M)	100/100 (90/93)	pRE25, pAMβ1	
59	50935	51183	F	249	82	33.9	*copS*	55/78	pAMβ1, pIP501, pRE25	copy number repressor
60	51575	53065	F	1491	496	35.5	RepE, RepS	98/99	pAMβ1, pIP501, pRE25	replication protein
61	53413	53583	F	171	56	30.7	orfα	100/100 (94/98)	pRE25, pDB101	
62	53597	54214	F	618	205	35.5	resolvase	94/98	pAMβ1	resolvase
63	54214	56358	F	2145	714	35.8	topoisomerase	97/98	pAMβ1	relaxase
64	56506	56745	F	240	79	33.7	omega2	98/100	pIlo8 (derivative of pIP501)	pIlo8 is a deletion derivative of pIP501
65	56794	56877	F	84	27	29.5	orf13, *erm* (C)	100/100	pRE25	MSL leader peptide
66	57002	57739	F	738	245	33.1	orf14, *erm* (B)	100/100	pRE25	erythromycin resistance protein
67	57744	57875	F	132	43	38.3	orf15 (protein M)	100/100 (90/93)	pRE25, pAMβ1	
68	57876	58169	F	294	97	32.5	truncated topoisomerase	88/89	pRE25	truncated topoisomerase
69	58272	58829	F	558	185	32.5	truncated ATPase	100/100	pRE25	truncated ParA family protein
70	59080	59565	R	486	161	28.6	*vanZ*	100/100	Tn*1546*	
71	59718	60629	R	912	303	34.1	*vanY*	100/100	Tn*1546*	
72	61057	61665	R	609	202	43.8	*vanX*	100/100	Tn*1546*	
73	61671	62702	R	1032	343	44.7	*vanA*	100/100	Tn*1546*	
74	62695	63663	R	969	322	44.5	*vanH*	100/100	Tn*1546*	
75	63878	65032	R	1155	384	41.5	*vanS*	99/99	Tn*1546*	three amino acid substitution
76	65010	65705	R	696	231	41.5	*vanR*	100/100	Tn*1546*	
77	65919	66494	R	576	191	33.8	ORF2	100/100	Tn*1546*	
78	66640	69606	F	2967	988	37.3	ORF1	100/100	Tn*1546*	
79	69681	70024	F	344	113	30.9	truncated ATPase	97/100	pRE25	truncated ParA family protein
80	70116	70331	F	216	71	33.6	omega2 protein	98/100	pIlo8 (derivative of pIP501)	transcriptional repressor
81	70349	70621	F	273	90	29.9	orf8	100/100	pRE25	epsilon-zeta antitoxin 2
82	70623	70700	F	78	26	32.4	truncated zeta toxin	100/100	pRE25	zeta toxin, postregulational killing system
83	70754	71440	R	687	228	38.5	transposase	88/93	IS*1216*	transposase
84	71509	72302	F	794	263	37.6	truncated zeta toxin	100/100	pRE25	zeta toxin, postregulational killing system
85	72433	72723	F	291	96	32.3	orf21	97/98	pRE25	DnaJ-like protein
86	73006	73602	R	597	198	35.3	transcriptional regulator	67/85	*E. faecium DO*	TetR family transcriptinal regulator
87	73913	74794	F	882	293	39.7	ABC transporter	74/86	*E. faecium DO*	ABC transporter ATP-binding protein
88	74818	76431	F	1614	537	40.7	permease	58/77	*E. faecium DO*	Amino acid permeases, polyketide antibiotics
89	76503	77014	R	512	169	37.7	truncated transposase	42/62	IS*30*	truncated transposase
90	77015	77393	F	279	125	38.0	truncated zeta toxin	99/100	pRE25	truncated and frame-sihfted zet toxin
91	77567	79195	R	1629	542	30.4	transposase	49/65	IS*4*	transposase
92	79296	79451	F	156	51	31.7	truncated hypothetical protein	80/92	*Clostridium beijerincki*	truncated hypothetical protein
93	79516	80154	F	639	212	31.1	lactamase B	72/86	*Clostridium beijerincki*	Lactamase B metallo-β-lactamase superfamily
94	80315	81943	R	1629	542	30.3	transposase	48/65	IS*4*	transposase
95	82437	82754	F	318	105	31.4	orf20	66/80	pRE25	ssDNA binding protein
96	82765	83254	F	480	163	31.4	truncated orf21	99/100	pRE25	truncated DnaJ-like protein
97	83323	84009	F	687	228	36.4	mutated transposase	90/94	IS*1216*	transposase with non-sense mutation
98	84328	85014	F	687	228	37.3	transposase	100/100	IS*1216*	transposase

[a] Nine ORFs from ORF59 to ORF67, predicted as an integrated small plasmid shown by a thick red squar line with gray background on the map, were lacking in pTW24 (type B plasmid, expected as 77,792bp in size).

[b] pJM01 (*E. faecalis*) BAC^{r} TET^{r}, isolated from a dog with mastitis , New Zealand [50, 52].

[c] pRE25 (*E. faecalis*) CHL^{r} MLS^{r} (resistance to macrolide-lincosamide-streptogramin) Conjuagative plasmid, 50 kb, isolated from dry sausage, beef and pork, Europe [57].

[d] Bacitracin (BAC) is an antimicrobial that comprises a mixture of high-molecular-weight polypeptides produced by the organism *Bacillus licheniformis*. Bacitracin is used widely in topical applications in human medicine, and its oral use for the control of VRE has been suggested. Bacitracin is also used extensively for prophylaxis and therapy in food animals, particularly in broiler chicken production.
